# Supplementary material for: Characteristics and Outcomes of Bloodstream Infections in a Tertiary-Care Pediatric Hematology–Oncology Unit: A 10-Year Study
Source: J Clin Med. 2022 Feb 8;11(3):880. doi: 10.3390/jcm11030880 (PMC8836920; doi:10.3390/jcm11030880)
Supplement: Supplementary file 1 [file jcm-11-00880-s001.zip › jcm-1546790-supplementary.pdf]

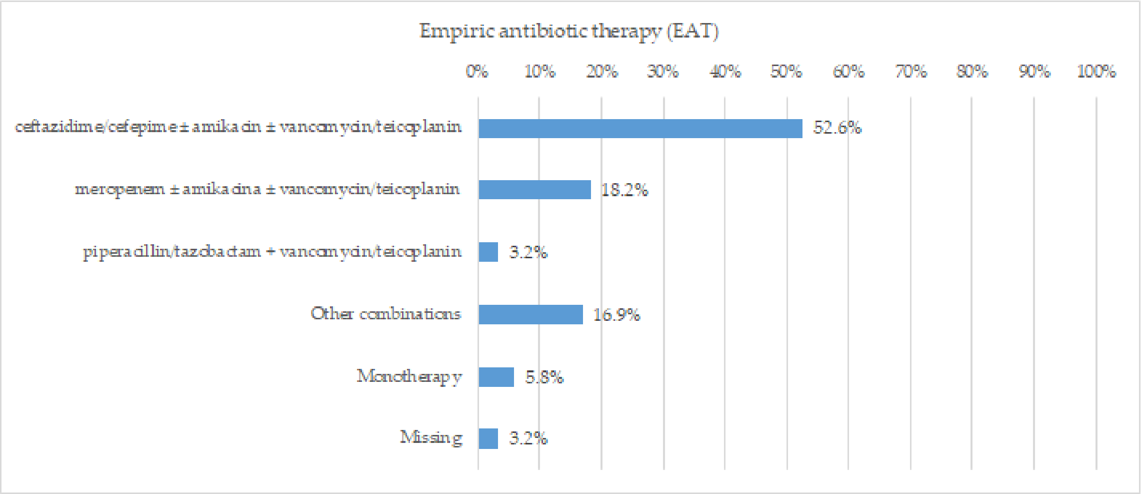

Figure S1. Empiric antibiotic therapy.

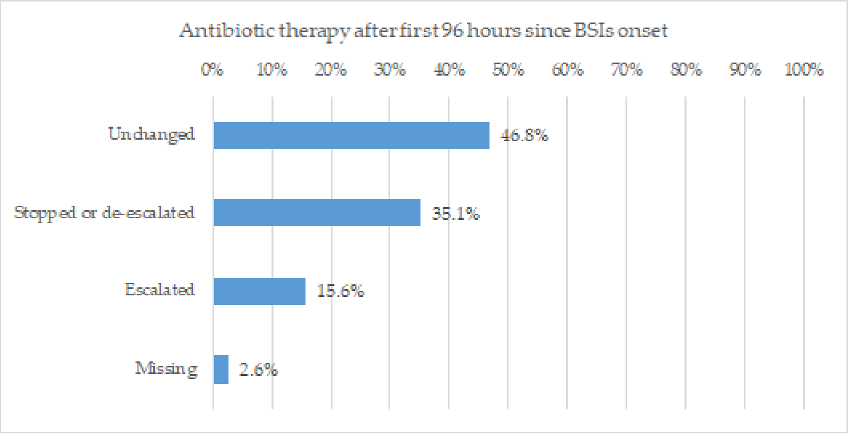

Figure S2. Antibiotic therapy changes that occurred after the first 96 h since the onset of bloodstream infections.
